# Oxaliplatin-induced haematological toxicity and splenomegaly in mice

**DOI:** 10.1371/journal.pone.0238164

**Published:** 2020-09-02

**Authors:** Justin G. Lees, Daniel White, Brooke A. Keating, Mallory E. Barkl-Luke, Preet G. S. Makker, David Goldstein, Gila Moalem-Taylor

**Affiliations:** 1 School of Medical Sciences, The University of New South Wales, Sydney, New South Wales, Australia; 2 Prince of Wales Clinical School, The University of New South Wales, Sydney, New South Wales, Australia; University of Kentucky, UNITED STATES

## Abstract

**Purpose:**

Haematological toxicities occur in patients receiving oxaliplatin. Mild anaemia (grade 1–2) is a common side effect and approximately 90% of recipients develop measurable spleen enlargement. Although generally asymptomatic, oxaliplatin-induced splenomegaly is independently associated with complications following liver resection for colorectal liver metastasis and separately with poorer patient outcomes. Here, we investigated oxaliplatin-induced haematological toxicities and splenomegaly in mice treated with escalating dosages comparable to those prescribed to colorectal cancer patients.

**Methods:**

Blood was analysed, and smears assessed using Wright-Giemsa staining. Paw coloration was quantified as a marker of anaemia. Spleen weight and morphology were assessed for abnormalities relating to splenomegaly and a flow cytometry and multiplex cytokine array assessment was performed on splenocytes. The liver was assessed for sinusoidal obstructive syndrome.

**Results:**

Blood analysis showed dose dependent decreases in white and red blood cell counts, and significant changes in haematological indices. Front and hind paws exhibited dose dependent and dramatic discoloration indicative of anaemia. Spleen weight was significantly increased indicating splenomegaly, and red pulp tissue exhibited substantial dysplasia. Cytokines and chemokines within the spleen were significantly affected with temporal upregulation of IL-6, IL-1α and G-CSF and downregulation of IL-1β, IL-12p40, MIP-1β, IL-2 and RANTES. Flow cytometric analysis demonstrated alterations in splenocyte populations, including a significant reduction in CD45+ cells. Histological staining of the liver showed no evidence of sinusoidal obstructive syndrome but there were signs suggestive of extramedullary haematopoiesis.

**Conclusion:**

Chronic oxaliplatin treatment dose dependently induced haematological toxicity and splenomegaly characterised by numerous physiological and morphological changes, which occurred independently of sinusoidal obstructive syndrome.

## Introduction

Oxaliplatin is a 3^rd^ generation platinum-based chemotherapeutic commonly administered in conjunction with leucovorin and fluorouracil (FOLFOX) for neoadjuvant and adjuvant treatment of colorectal cancer (CRC) [1]. A range of side effects are associated with oxaliplatin including fatigue, nausea, peripheral neuropathy and haematological toxicity [2]. Addition of oxaliplatin to fluorouracil and leucovorin for the treatment of CRC patients causes a significant increase in all grade anaemia when compared to fluorouracil and leucovorin treatment alone [1]. CRC patients also often have pre-operative cancer-related anaemia [3], which is reported to be a factor in poor disease progression and recovery [4]. Generally, however, anaemia is relatively mild with most patients experiencing grade 1–2 and only a small percentage of patients experiencing grade 3–4 [1]. Additionally, there are case reports that describe oxaliplatin-induced immune-mediated haemolytic anaemia [5].

More recently, it has been demonstrated that approximately 90% of patients develop enlarged spleens [6], with splenomegaly (defined as ≥50% increase in spleen size) present in approximately 25% of oxaliplatin recipients [7]. Prevalence is highly correlated with dosage and up to 67% of patients receiving ≥12-cycles of the FOLFOX regimen reportedly experienced splenomegaly [8]. Spleen size typically returned to normal between 3–12 months following treatment completion [7]. However, in a substantial patient population, spleen enlargement persisted for greater than 12 months [9]. The cause of splenomegaly in oxaliplatin-treated patients remains unclear and indeed there may be multiple contributing factors, with portal hypertension due to hepatic sinusoidal obstructive syndrome (SOS) [10] and haematological toxicity implicated. Moderate to severe SOS was identified in more than 50% of patients receiving oxaliplatin prior to liver resection for colorectal cancer liver metastasis (CRLM), whereas no SOS was evident in CRLM patients who had liver resection without prior oxaliplatin treatment [11]. Portal hypertension resulting from SOS could therefore account for spleen enlargement [12], but a recent large scale clinical study indicated too few events of sinusoidal injury to draw a correlation with spleen volume increase [13].

In relation to cancer progression and cancer-related complications, the effects and outcomes resulting from splenomegaly are poorly understood. There is some evidence to suggest that splenomegaly can potentially be used as a biomarker for the incidence of SOS [7]. Furthermore, inclusion of Bevacizumab in the treatment regimen can reduce the incidence of splenomegaly and SOS, but the precise reason for this outcome is unknown [10, 14]. Interestingly, baseline spleen enlargement prior to oxaliplatin treatment is associated with poor therapeutic response in patients with advanced pancreatic cancer [15] and splenomegaly is an independent predictor of complications in CRLM patients [13]. In this study, we addressed the gap in knowledge relating to oxaliplatin-induced anaemia and splenomegaly [16], by using a preclinical mouse model to investigate the effects of different oxaliplatin regimens on blood composition, spleen size, immune cell phenotype and cytokine profile in the spleen, and liver histology.

## Materials and methods

### Animals

Experiments were carried out on 8-12-week-old male C57BL/6J and BALB/c mice (Animal Resource Centre, Moss Vale, NSW, Australia). Animals were housed with free access to food and water and maintained on a 12:12-hour light/dark cycle. From the time of arrival to the time of euthanasia, mice were observed daily for any signs of ill health. During the treatment period, comprehensive health checks including weighing mice, assessing coats condition, skin condition and mobility were also conducted at least four times a week. Chemotherapy treatment was well tolerated by mice and there was no treatment related adverse events. At the conclusion of experimental period, mice were deeply anesthetised in 5% isoflurane in O_2_ gas. Following dissection of spleen, liver and blood collection as described below, mice were euthanised by rapid decapitation or lethal intraperitoneal injection of Lethabarb (Pentobarbitone Sodium 100mg/kg). All experiments were performed in accordance and with approval of The University of New South Wales—Animal Care and Ethic Committee (15/141B and 16/111B).

### Oxaliplatin regimen

Oxaliplatin (O9512-5MG; Sigma-Aldrich, St Louis, MO) was dissolved in 5% dextrose/water to a concentration of 1mg/ml. Mice received 2.5 mg/kg intraperitoneal injections of oxaliplatin. For the highest dose chronic regimen, mice were injected on four consecutive days (Mon-Thurs), over three consecutive weeks. In total, these mice received 12 injections between ‘Day 0’ and ‘Day 17’ and a cumulative dose of 30 mg/kg. For the medium dose regimen, mice received 9 injections on three consecutive days (Mon-Wed), over three consecutive weeks between ‘Day 0’ and ‘Day 16’ and a cumulative dose of 22.5 mg/kg. For the lowest dose regimen mice received the first six injections of the highest dose protocol between ‘Day 0’ and ‘Day 8’ and a cumulative dose of 15 mg/kg. Control group mice received 5% dextrose/water injections.

### Blood analysis and Wright-Giemsa blood smear staining

A 27-gauge needle attached to a 1ml syringe was used to extract blood from the heart of isoflurane-anaesthetised mice. Approximately 100μl of whole blood was added to an Eppendorf containing 10μl of the anticoagulant ACD-B. Within 5 hours blood was analysed using a Haematology Coulter Ac.T Diff. (Beckman Coulter) equipped with a veterinary card for assessment of small animal blood. From the remaining blood sample within the syringe, a drop was placed directly onto a slide to make a smear. Smears were air dried and then fixed in methanol for 3 minutes at room temperature. Air dried slides were then stained with Wright-Giemsa (Sigma-Aldrich) and subsequently imaged using a BX-51 light microscope (Olympus, Tokyo, Japan).

### Assessment of paw discolouration

Mice were placed on a suspended transparent glass platform and habituated to their environment. Once habituated and at rest, photos of their front and hind paws were captured from underneath the platform. For consistency, all photos were captured with a single DSLR camera (Canon 7D; Canon, Tokyo, Japan) using a fixed contrast, aperture, focal length, white-balance, exposure and distance from the feet. A section of each image containing only the foot-pad of the hind or front paws was selected and analysed using the Image-J ‘Fiji’ software. This freeware java-based image software, developed by the National Institute of Health (NIH), is commonly used to obtain quantitative data from visual results such as the bands on Western blot patterns [17]. Using this software, red pixels within the image were isolated, and a threshold for their ‘darkness’ was set and kept constant between each image. Darker/redder pixels fell within this threshold, whilst whiter pixels did not. Following this, the percentage area of the photo sample containing darker/redder pixels was calculated. Using the intensity of red pixels in this way, we were able to quantify the presence of pallor or plethora within the hind/front paws of these mice. Values from the left and right paws were averaged to get an individual front or hind paw value for each mouse assessed. Images were taken within 24 hours of the ‘Day 30’ euthanising time point. A similar method of analysis has been conducted using this software and pixel-thresholding for clinical quantification of conjunctival pallor between haemato-oncology in- and out-patients [18].

### Spleen and liver histology

Mice were anesthetised under 5% isoflurane. The spleen and liver were dissected and fixed in 4% paraformaldehyde. Prior to processing, spleens were blotted dry and weighed. Spleen and liver were processed overnight using an Excelsior^™^ AS tissue processor (Thermo-fisher, Waltham, MA), embedded in paraffin and sectioned using a microtome (4μm sections). Sections were stained with haematoxylin and eosin and subsequently imaged using a BX-51 light microscope (Olympus).

### Cytokine/Chemokine multiplex assay

Under 5% isoflurane anaesthesia, spleens were dissected and snap frozen in liquid nitrogen for storage at -80°C. Spleens were thawed on ice and weighed, protein was extracted from ~20mg of spleen tissue per 100μl of protein extraction lysis buffer. Tissue was homogenised using a Precellys-Cryolys (Bertin Instruments, Montigny-le-Bretonneux, France). Lysate was then centrifuged, and supernatant stored at -80°C prior to analysis. The Bio-plex 23 cytokine/chemokine mouse array bead kit was used for cytokine/chemokine quantification following the manufacturer’s instructions (Bio-Rad, Hercules, CA) using protein from 4mg of spleen tissue per mouse. The beads were analysed using a MAGPIX system (Luminex, Austin, TX) and the medium fluorescent intensity values (corrected for background reading) were compared.

### Flow cytometry

Animals were anaesthetised under 5% isoflurane, and spleens were removed and placed in phosphate buffered saline (PBS) on ice. Prior to processing, spleens were blotted dry and weighed. Spleens were mechanically ground through a 40μm cell strainer (Thermo-Fischer) in 10mL PBS to obtain a single cell suspension of mononuclear cells. Samples were then centrifuged for 5 minutes at 4°C at 600x*G*, and the supernatant discarded. Spleens were resuspended in 5mL red blood lysis buffer and agitated at room temperature for 5 minutes. 10mL PBS was then added to all samples, before centrifuging at 600x*G* for 5 minutes at 4°C. The supernatant was discarded, and spleen cells were resuspended in 7mL PBS. 100μL of cells were transferred to 1.5mL tubes and incubated with 1μL/sample Zombie cell viability dye (BioLegend, San Diego, CA, USA) for 30 minutes in the dark at room temperature. 1mL autoMACS running buffer (Miltenyi Biotec, Bergisch Gladbach, Germany) was then added to samples before centrifuging at 600x*G* for 5 minutes at 4°C and discarding the supernatant. Cell surface markers were stained for 30 minutes at 4°C using the following combinations of antibodies (0.5μL of each unless otherwise specified): anti-CD45-BV510 (BioLegend; 103138, Lot B295493), anti-CD11b-BV650 (BioLegend; 101239, Lot B251560), anti-CD4-BV711 (BioLegend; 100557, Lot B256706), anti-CD25-APC (eBioscience; 17-0251-82, Lot 4329682), anti-CD8a-BV421 (BioLegend; 100753, Lot B284314), anti-CD19-PEDazzle (BioLegend; 115553, Lot B272775), anti-CD11c-PE (BioLegend; 117308, Lot B278350). 1mL autoMACS running buffer was then added to all samples, which were centrifuged for 5 minutes at 4°C at 600x*G* and supernatant discarded, repeated twice more. Samples were then incubated overnight at 4°C with fixation/permeabilisation buffer (Thermo-Fisher) (1mL/sample enabling intracellular staining for a separate unpublished study). Following this, samples were washed twice in 1 x permeabilisation buffer (Thermo-Fisher) for 30 minutes at room temperature in the dark. Samples were then washed three times in 1 x permeabilisation buffer (600x*G*, 5 minutes, 4°C), and resuspended in autoMACS running buffer to a total volume of 200μL per sample. Cytometric analysis was performed using an LSRFortessa X20 flow cytometer (Becton Dickinson). Results were analysed using FACSDiva (Becton Dickinson, Franklin Lakes, NJ) and FlowJo software (FlowJo, Ashland, OR). Fluorescence minus one (FMO) controls were included to help define positive populations of cells for a given antibody.

### Statistical analysis

Statistical analysis was performed using Prism Version 7 (Graph Pad Software, San Diego, CA). Data acquired from blood analysis, paw coloration, spleen weight, FACS analysis and cytokine/chemokine assay were tested for normal distribution using the Shapiro-Wilk normality test (α = 0.05). If data was normally distributed, One-way Analysis of Variance (ANOVA) was used for comparison of more than two groups with multiple comparison correction performed using Dunnett test, and Student’s t-tests was used for comparing two groups. If data was not normally distributed, then Kruskal-Wallis test was used for comparison of more than two groups with multiple comparison correction performed using Dunn’s test and Mann-Whitney was used for comparing two groups. For all ANOVA tests, each treatment group was compared to the control group and significant adjusted p-values are reported. For the cytokine/chemokine assay, ‘Benjamini and Hochberg’ false discovery rate analysis on the ranked p-values was performed and the resulting significant adjusted p-values (q-values) are reported. In all cases, a p or q-value of less than 0.05 was considered statistically significant.

## Results

### Chronic oxaliplatin treatment dose-dependently induces significant changes in haematological indices

**Fig 1:**
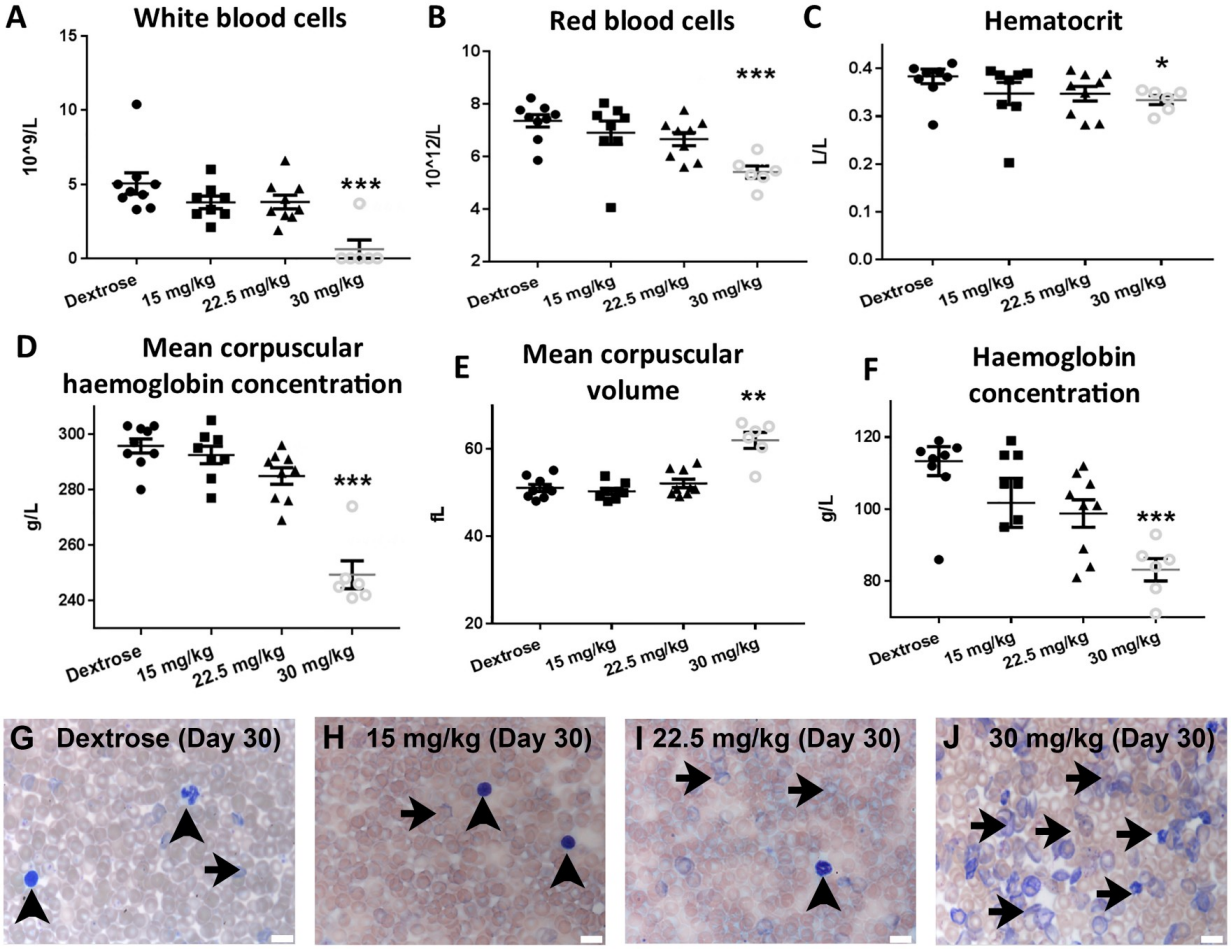
Oxaliplatin induces dose-dependent changes in haematological measures. Dot plots of blood analysis on ‘Day 30’ after oxaliplatin treatment in C57BL6/J mice for (A) White blood cells (WBC), (B) Red blood cells (RBC), (C) Hematocrit, (D) Mean corpuscular haemoglobin concentration (MCHC), (E) Mean corpuscular volume (MCV), (F) Haemoglobin concentration. (G,H,I,J) Brightfield images of Wright-Giemsa blood smears; arrowheads—nucleated leukocytes, arrows–denucleated cells. (Mean ± SEM; Dextrose n = 9, 15 mg/kg n = 8, 22.5 mg/kg n = 9, 30 mg/kg n = 6; p<0.05 = *, p<0.01 = **, p<0.001 = ***; scale bar = 10μm).

Mice were treated with three regimens of systemic injections of oxaliplatin with cumulative dosages of 15 mg/kg, 22.5 mg/kg and 30 mg/kg. At 30 days following first injection (‘Day 30’), whole blood was assessed using an ‘Ac.T Diff’ blood analyser. Oxaliplatin induced dose-dependent and highly significant changes in the cellular composition of blood. Specifically, in mice treated with the 30 mg/kg dosage there was a significant reduction in white blood cells (WBC), red blood cells (RBC), mean corpuscular haemoglobin concentration (MCHC), and haemoglobin concentration, accompanied by a significant increase in mean corpuscular volume (MCV) (Fig 1A–1F). There was also a noticeable dose-dependent non-significant trend in these measures in mice treated with 15 mg/kg and 22.5 mg/kg cumulative dosages (Fig 1D and 1F).

Blood smears stained with Wright-Giemsa at ‘Day 30’ indicated a reduction in the presence of nucleated leukocytes in the 30 mg/kg dosage group (Fig 1J), which was consistent with the ‘Ac.T Diff’ WBC analysis (Fig 1A). Nucleated leukocytes were clearly present in the control and 15 mg/kg and 22.5 mg/kg treatment groups (Fig 1G–1I - arrowheads). Abnormal staining of denucleated cells was evident in smears from oxaliplatin-treated mice and particularly in mice treated with the 30 mg/kg dosage (Fig 1J—arrows). The denucleated cells appear to represent polychromatophilic reticulocytes and spherocytes. These results indicate that oxaliplatin affects both WBC and RBC, and at a high cumulative dose (30 mg/kg) it induces the formation of dysfunctional RBC and macrocytic anaemia.

**Fig 2:**
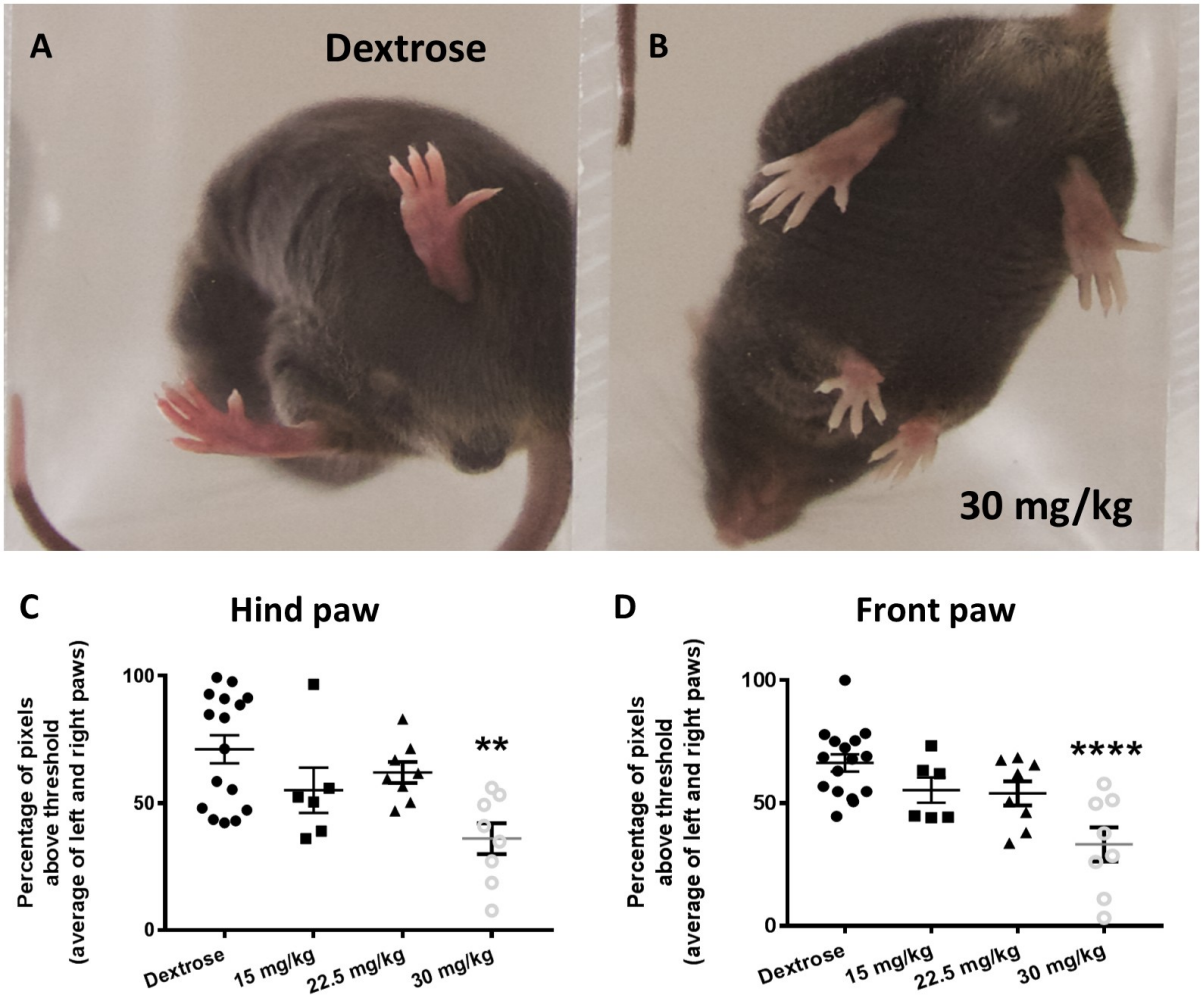
Hind and front paw coloration. Pixel threshold technique was used to quantify coloration of C57BL6/J mouse paws assessing images taken within 24 hours of ‘Day 30’ tissue collection time point. Example images of (A) control dextrose treated and (B) 30 mg/kg oxaliplatin treated mice used for threshold quantification of paw colour. Dot plots of (C) Hind and (D) Front paw quantification of pixel threshold for different treatment groups (a higher percentage equates to redder paws, whereas lower percentage equates to whiter paws) (Mean ± SEM; Dextrose n = 16, 15 mg/kg n = 6, 22.5 mg/kg n = 8, 30 mg/kg n = 8; p<0.01 = **, p<0.0001 = ****).

In support of this, a secondary assessment of anaemia was used by quantifying the whitening (discoloration) of front and hind paws of mice treated with different dosages of oxaliplatin. Interestingly, we observed very significant whitening of both front and hind paws in the mice treated with 30 mg/kg dosage. This was quantified using a pixel threshold technique from images captured within 24 hours of the ‘Day 30’ tissue collection time point (Fig 2A–2D).

### Chronic oxaliplatin treatment results in dose-dependent splenomegaly

**Fig 3:**
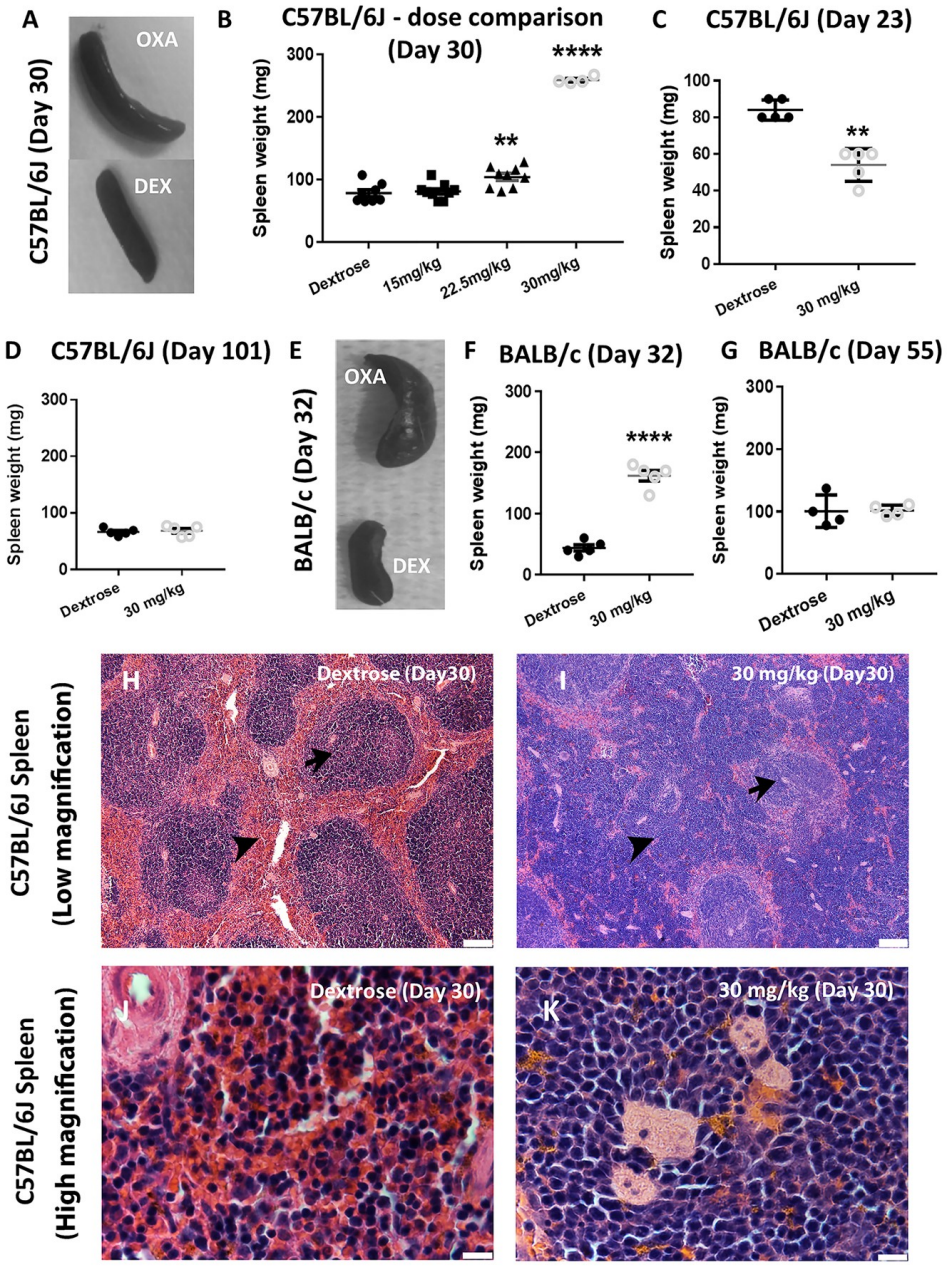
Oxaliplatin induces dose-dependent splenomegaly. (A) C57BL/6J mice spleens from dextrose treated and oxaliplatin 30 mg/kg treated mice. (B) Weight of C57BL/6J mice spleens treated with escalating oxaliplatin dosage regimens demonstrated significant spleen weight increase at ‘Day 30’ (Mean ± SEM; Dextrose n = 8, 15 mg/kg n = 9, 22.5 mg/kg n = 9, 30 mg/kg n = 4; p<0.01 = **, p<0.0001 = ****), (C) In mice treated with 30 mg/kg regimen the spleen weight was reduced at ‘Day-23’ (Mean ± SEM; Dextrose n = 5, 30 mg/kg n = 5; p<0.01 = **), (D) At 3 months (101 days) C57BL/6J spleen weight had returned to normal (Mean ± SEM; Dextrose n = 5, 30 mg/kg n = 5), (E-F) BALB/c mice treated with chronic oxaliplatin regimen demonstrated significant spleen weight increase at ‘Day-32’ (Mean ± SEM; Dextrose n = 5, 30 mg/kg n = 5; p<0.0001 = ****), (G) At 55 days BALB/c spleen weight had returned to normal (Mean ± SEM; Dextrose n = 4, 30 mg/kg n = 4). BALB/c strain data are related to (Fig 3E-3G) only, all other data in this manuscript relate to C57BL/6J mice. Histology of the spleen from (H, J) Dextrose treated control C57BL/6J mice and (I, K) C57BL/6J mice treated with 30 mg/kg dosage at ‘Day-30’; arrowheads—red pulp, arrows–white pulp; J,K = red pulp region. (H, I scale bar = 100μm; J, K scale bar = 10μm).

Splenomegaly presents as a common haematological clinical outcome following oxaliplatin treatment, with ~90% of patients exhibiting signs of spleen enlargement [6]. Considering splenomegaly is clinically dependent on dosage [7, 8], spleen weight was assessed in mice receiving escalating dosages of oxaliplatin. In C57BL6/J mice, there was a dose-dependent increase in spleen weight that reached a significant level at ‘Day 30’ for both 22.5 mg/kg and 30 mg/kg cumulative dosage groups (Fig 3A and 3B). Curiously, there have been previous reports of splenic atrophy following similar oxaliplatin dosage in mice at ~21 days after first injection [19]. A separate cohort of mice receiving the 30 mg/kg dosage were culled at the earlier time point of ‘Day 23’, and in agreement with these previous studies the spleen weight was significantly reduced at this time point (Fig 3C) [19]. As splenomegaly generally resolves in patients following an interval after treatment completion [7], we assessed mice treated with the 30 mg/kg dosage at three months (101 days after 1^st^ injection). In these mice, we observed a normalisation of spleen weight (Fig 3D). Furthermore, it is well known that substantial differences exist between mouse strains in relation to how they tolerate toxic insults [20]. A cohort of BALB/c mice were treated with the 30 mg/kg dosage regimen and spleen weight was assessed at ‘Day 32’. In line with results for the C57BL/6J strain, there was a highly significant increase in spleen weight in BALB/c mice (Fig 3E and 3F), this spleen enlargement was resolved when checked at day 55 after first injection (Fig 3G). The varied timing of endpoints between mouse strains was due to unintended logistical reasons. These two mouse strains have been commonly utilised in preclinical studies investigating the effects of oxaliplatin [21]. The results indicate dose-dependent oxaliplatin-induced splenomegaly occurs following a period of splenic atrophy. Oxaliplatin induces splenomegaly in two separate mouse strains and this condition resolves following an interval after cessation of treatment, in agreement with reported clinical findings [7, 8].

### Chronic oxaliplatin treatment causes changes in spleen histology

Hematoxylin and eosin staining of splenic tissue from the vehicle-treated control mice depicted a normal morphology, with a clearly defined white and red pulp region (Fig 3H and 3J). However, enlarged spleens from mice treated with the 30 mg/kg dosage at ‘Day 30’ displayed a distinctly irregular histology, whereby there was loss of follicular structure and dysplasia. This was predominantly in the red pulp (Fig 3I—arrowhead and Fig 3K).

### Chronic oxaliplatin treatment causes changes in spleen cytokine/chemokine profile

**Fig 4:**
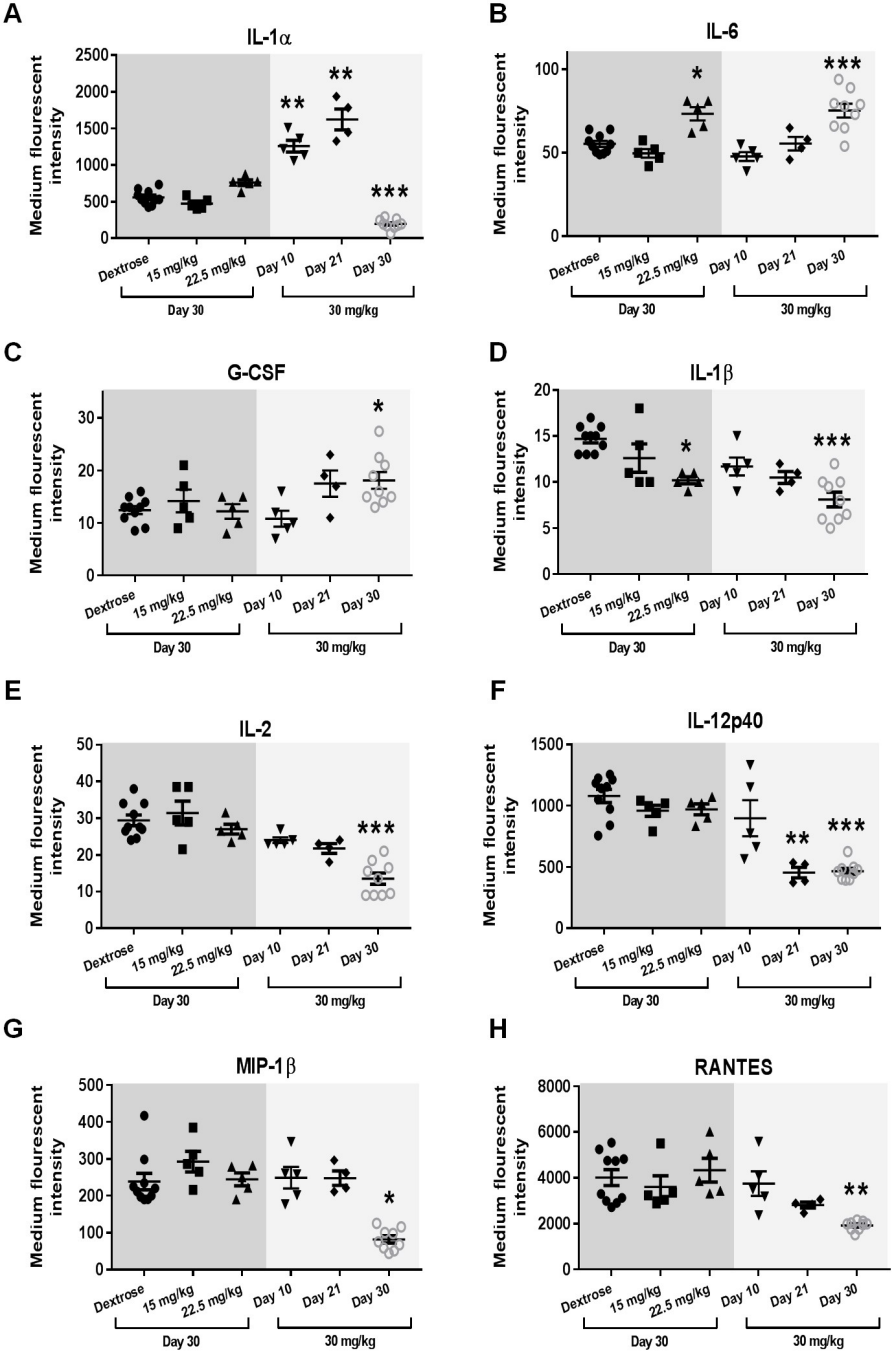
Splenic expression of cytokines and chemokines in oxaliplatin-treated mice. Dot plots of cytokine and chemokine analysis in spleens of C57BL6/J mice for (A) IL-1α, (B) IL-6, (C) G-CSF, (D) IL-1β, (E) IL-2, (F) IL-12p40, (G) MIP-1β and (H) RANTES (Mean ± SEM; Dextrose n = 10, 15 mg/kg n = 5, 22.5 mg/kg n = 5, 30 mg/kg (Day 10) n = 5, 30 mg/kg (Day 21) n = 4, 30 mg/kg (Day 30) n = 9; q<0.05 = *, q<0.01 = **, q<0.001 = ***).

A multiplex assay capable of detecting 23 individual cytokines and chemokines was undertaken on protein extracted from splenic tissue on ‘Day 30’ following different oxaliplatin cumulative doses, and at different days following treatment with the high dose regimen (30mg/kg) (Fig 4A–4H). Intriguingly, IL-1α expression at ‘Day 10’ and ‘Day 21’ of the 30 mg/kg dosage regimen was significantly elevated, whilst at ‘Day 30’ it was significantly reduced (Fig 4A). IL-6 expression was significantly elevated on ‘Day 30’ of both the 22.5 mg/kg and 30 mg/kg regimens (Fig 4B) and G-CSF was significantly elevated on ‘Day 30’ of the 30 mg/kg regimen (Fig 4C). IL-1β, IL-12p40, MIP-1β, IL-2, and RANTES were all significantly reduced on ‘Day 30’ of 30 mg/kg regimen (Fig 4D–4H). Furthermore, IL-1β was significantly reduced at ‘Day 30’ of the 22.5 mg/kg regimen (Fig 4D), whilst IL-12p40 was reduced at ‘Day 21’ of the 30 mg/kg regimen (Fig 4F). These changes in cytokine and chemokine expression levels are consistent with substantial oxaliplatin-induced alteration of spleen physiology.

### Chronic oxaliplatin treatment causes changes in spleen immune cell profile

**Fig 5:**
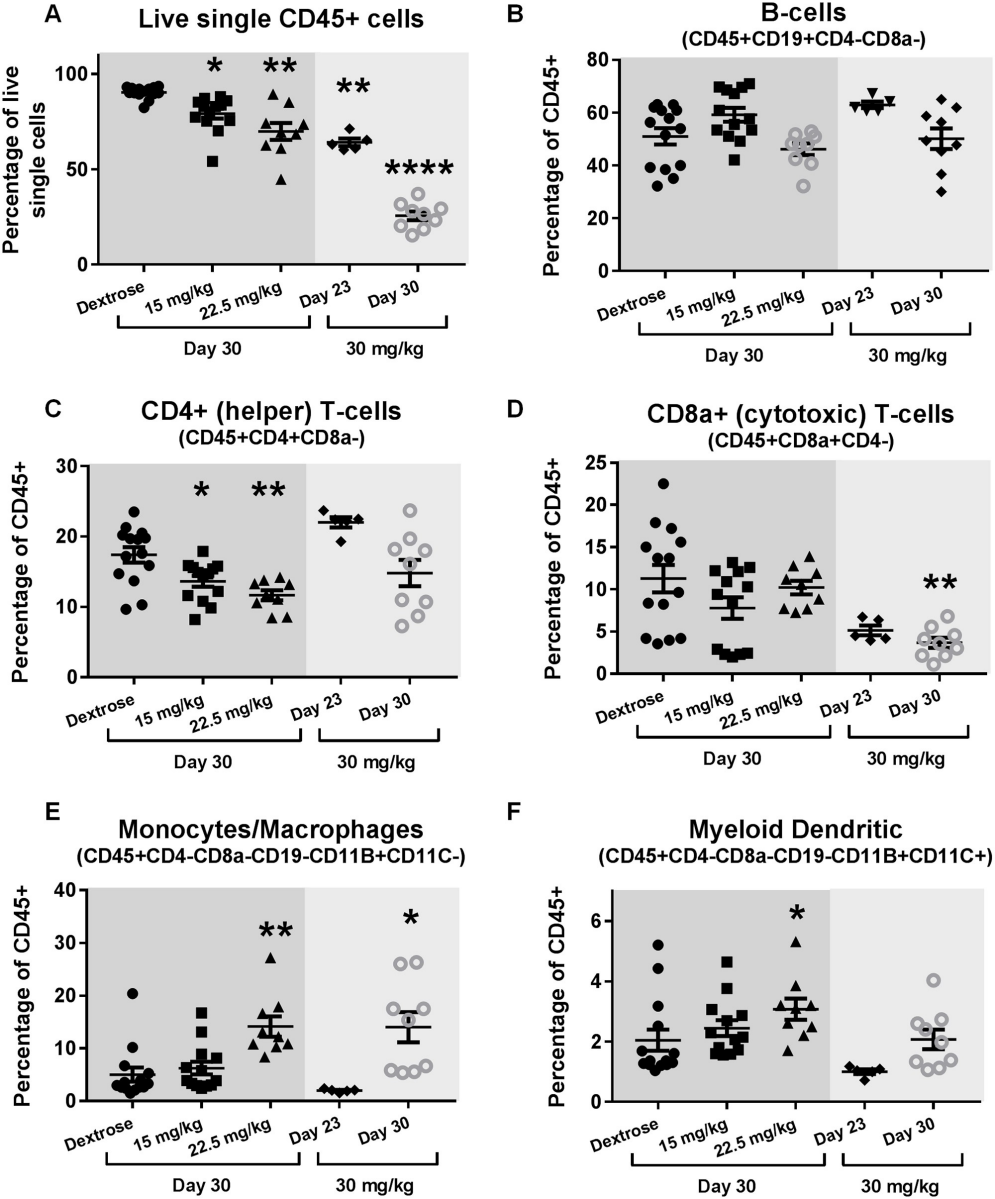
Oxaliplatin substantially alters splenocyte composition. Dot plots of FACS analysis of spleens from C57BL/6J mice for (A) Live CD45+ single cells, (B) B-cells (CD45+CD19+CD4-CD8a-), (C) CD4+ (helper) T-cells (CD45+CD4+CD8a-), (D) CD8a+ (cytotoxic) T-cells (CD45+CD8a+CD4-), (E) Monocytes/Macrophages (CD45+CD4-CD8a-CD19-CD11B+CD11C-), (F) Myeloid dendritic (CD45+CD4-CD8a-CD19-CD11B+CD11C+) (Mean ± SEM; Dextrose n = 14, 15 mg/kg n = 13, 22.5 mg/kg n = 9, 30 mg/kg (Day 23) n = 5, 30 mg/kg (Day 30) n = 9; p<0.05 = *, p<0.01 = **, p<0.0001 = ****).

Flow cytometric analysis was undertaken in order to gain further insight into the cellular changes occurring in the spleen following oxaliplatin treatment. The proportion of live cells staining positive for pan-leukocyte marker CD45 was dose-dependently and significantly reduced in all oxaliplatin treatment groups compared to the controls (Fig 5A). With regards to the proportion of CD45+ cells in the lymphoid lineage, there was no significant change in B-Cells (Fig 5B) and only a significant reduction of CD4+ (helper) T-cells in the 15 mg/kg and 22.5 mg/kg regimen at ‘Day 30’ (Fig 5C). CD8a+ (cytotoxic) T-cells were significantly reduced at ‘Day 30’ in the 30 mg/kg regimen (Fig 5D). Within the myeloid lineage, there were significant increases in the proportion of monocytes/macrophages at ‘Day 30’ in both the 22.5 mg/kg and 30 mg/kg regimens (Fig 5E). Additionally, dendritic cells were significantly increased at ‘Day 30’ of the 22.5 mg/kg regimen (Fig 5F). Overall, the cellular composition of the spleen was drastically altered due to reduction in the proportion of CD45+ cells and altered immune cell profile within the CD45+ population following oxaliplatin treatment.

### No indication of SOS in chronic oxaliplatin-treated mice

**Fig 6:**
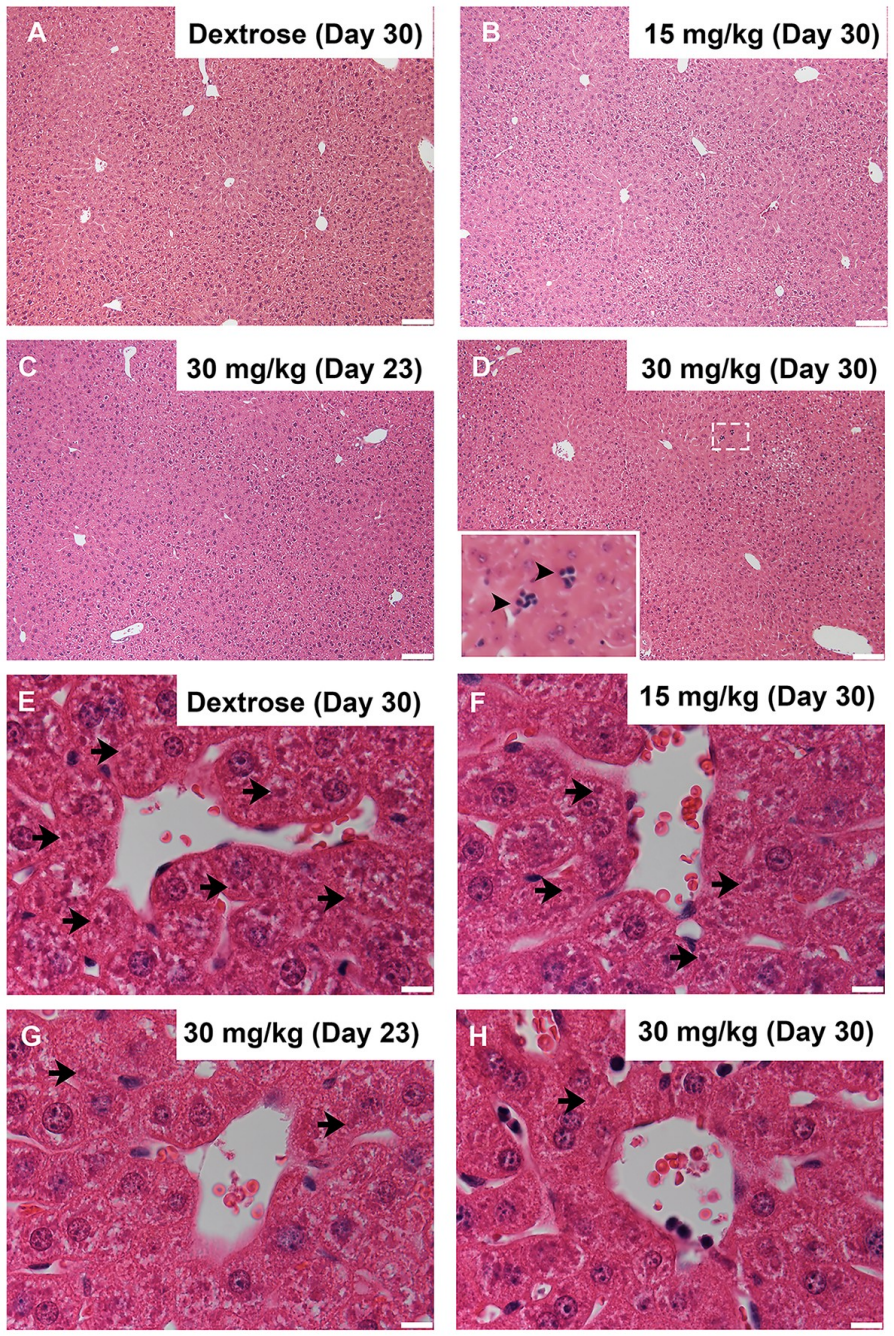
Histology of the liver in oxaliplatin-treated mice. Representative images of H&E histology of the liver of C57BL6J mice; (A, E) Dextrose, (B, F) 15 mg/kg, (C, G) 30 mg/kg ‘Day-23’ and (D, H) 30 mg/kg ‘Day-30’ groups; arrowheads = clustered cells, arrows–intracellular granules (A-D scale bar = 100μm; E-H scale bar = 10μm).

Clinical studies have implicated an association between splenomegaly and SOS in patients undergoing liver resection following preoperative oxaliplatin treatment for CRLM [10, 11]. It was therefore of interest to investigate the liver of oxaliplatin-treated mice, determining the incidence of SOS. Histological assessment of the gross structure of liver lobules did not indicate any obvious difference in morphology which would be indicative of SOS between the control group and 30 mg/kg dosage group at ‘Day 23’ and ‘Day 30’ or the 15 mg/kg dosage group at ‘Day 30’ (Fig 6A–6D). However, there were clusters of dark stained nucleated cells embedded throughout the liver tissue of the 30 mg/kg dosage group at ‘Day 30’ that were not present in any other treatment group (Fig 6D insert) and are characteristic of extramedullary haematopoiesis [22]. Observation of hepatocytes under high magnification also indicated less intracellular granular structures in oxaliplatin-treated mice (Fig 6E–6H). Overall, we found no definitive histological signs of SOS observed in any of the liver samples to suggest its role in the incidence of splenomegaly.

## Discussion

The findings of this study represent the first detailed characterisation of anaemia and splenomegaly following systemic treatment with oxaliplatin in a preclinical mouse model. Although severe anaemia is rare, the incidence of splenomegaly is relatively common in patients receiving oxaliplatin but has been largely ignored due to its asymptomatic nature. An exception is the CRLM patient group, where splenomegaly is associated with the occurrence of SOS and is proposed as a biomarker for increased occurrence of post-operative complications [7]. There is now evidence that pre-treatment anaemia and post-treatment splenomegaly in CRC patients may have a physiological impact on patient outcome and cancer progression [4, 15, 23]. Whilst some form of spleen enlargement is known to occur in >90% of patients [6], the mechanisms involved could have important implications regarding the anti-tumour activity of oxaliplatin and potential combination of this chemotherapeutic with immunotherapies, given the critical role of the immune system in tumour suppression.

Analysis of blood from oxaliplatin-treated mice at ‘Day-30’ indicated a dose-dependent reduced WBC count and macrocytic anaemia. Blood smears for the 30 mg/kg dosage group confirmed the reduced presence of nucleated WBC. Together, these data suggest significantly reduced circulating WBC, which could impact diverse physiological functions such as adaptive immune response. The reduced RBC count and elevated MCV characterises macrocytic anaemia and the blood smears demonstrate the presence of polychromatophilic reticulocytes or malformed RBC, such as spherocytes. Oxaliplatin is known to be toxic to RBC *in vitro* [24] and can interact directly with haemoglobin [25]. Indeed, it has recently been proposed that oxaliplatin interaction with haemoglobin in neuronal cells is a contributing factor to the development of oxaliplatin-induced peripheral neuropathy [26]. The lifespan of RBC in C57BL/6J mice is approximately 22 days [27], compared to approximately 120 days in humans. This fact should be considered when making translational comparisons and also may explain why it takes more than 20 days for splenomegaly to appear in these mice. It is likely that the lack of prior reporting of splenomegaly in preclinical studies using oxaliplatin treatment in mice is because many previous studies, including our own [28], have an earlier endpoint where splenomegaly would not have been apparent, or indeed the spleen is atrophied [19]. Here, we demonstrate that a cumulative dosage of 30 mg/kg caused consistent and highly significant (~300%) increase in spleen weight, which was observed in two separate mouse strains. In agreement with clinical findings, we demonstrate that the incidence of splenomegaly is dose dependent, with no significant increase in spleen weight in the lowest 15 mg/kg cumulative dosage group and an ~50% significant increase in spleen weight in the moderate 22.5 mg/kg cumulative dosage group.

Interestingly, and in agreement with previous findings we observed that the spleen in oxaliplatin-treated mice was significantly atrophied prior to enlargement [19]. The spleen is known to be a primary site for oxaliplatin damage and the red pulp dysplasia we observed has been previously reported in tumour-bearing mice receiving oxaliplatin, leucovorin and fluorouracil [29]. This curious biphasic progression suggests that oxaliplatin initially caused suppression of splenocytes and spleen shrinkage due to toxicity, and subsequently precipitated enlargement due to proliferation or infiltration of atypical cells.

Histological signs of extramedullary haematopoiesis were observed in the liver of the 30 mg/kg dosage group (Fig 6D insert). Extramedullary haematopoiesis is a mechanism utilised to salvage RBC production in both the spleen and the liver in periods of haematological toxicity and may be occurring in the spleen of these mice. Although extramedullary haematopoiesis is more commonly associated with small animals such as mice [30], extramedullary haematopoiesis does also occur in humans [31]. It is therefore possible that a component of the splenomegaly observed clinically in human patients may be related to this phenomenon of oxaliplatin-induced RBC toxicity.

The striking upregulation in the expression of the IL-1α pro-inflammatory cytokine at ‘Day 10’ and ‘Day21’ at the 30 mg/kg dosage could contribute mechanistically to the subsequent spleen enlargement. IL-1α is a powerful cytokine secreted in response to cellular stress [32] and specifically as an alarmin for DNA damage [33]. As DNA damage is a key sign of toxicity associated with oxaliplatin, elevated IL-1α expression may be a responding signal for immune cell DNA damage. It is also noteworthy that expression of this cytokine is significantly reduced in the ‘Day 30’ timepoint at the 30 mg/kg dosage. A logical interpretation would be that due to the extended period since cessation of treatment, cells initiating distress signals due to DNA damage are no longer present at ‘Day 30’. Alternatively, the corresponding occurrence of splenomegaly may induce down regulation of IL-1α signalling. Conversely, at ‘Day 30’, both G-CSF and IL-6 were elevated in the 30 mg/kg dosage group, and IL-6 was also elevated in the 22.5 mg/kg dosage group. G-CSF is a key factor that promotes the mobilisation of hematopoietic stem cells from the bone marrow [34]. As liver histological analysis indicated extramedullary haematopoiesis, this could also be occurring in the spleen where the high level of G-CSF may be responsible for attracting hematopoietic stem cells. In addition, G-CSF has been implicated in spleen enlargement [35]. IL-6, which is produced primarily by T-cells and macrophages, plays a key role in response to inflammation. IL-6 also has a well described role in haematopoietic function and expansion of haematopoietic progenitor cells [36]. RANTES (CCL5) was significantly reduced at ‘Day 30’ in the 30 mg/kg dosage group. Interestingly in CRLM patients RANTES is secreted by T-cells at the invasive margin of metastasis and promotes tumour growth [37].

With regards to the proportion of CD45+ cells, we found that at all time-points and dosages tested, there were significant changes in the spleens of oxaliplatin-treated mice. This was again dose-dependent, and while ~90% of single live cells were CD45+ in the control group, only ~25% of single live cells were CD45+ at ‘Day 30’ in the 30 mg/kg dosage group. However, it should be noted that the ‘Day 30’ mice had spleens that were ~4 times the weight of the control mice. Therefore, the total number of CD45+ cells within the spleens of both 30 mg/kg dosage group at ‘Day 30’ and control groups should be similar, and it is the substantial increase in CD45- cells which caused a significant proportional change and future studies will focus on characterising the nature of these CD45- cells. The most significant changes in CD45+ cells (by percentage) at ‘Day 30’ were a reduction in lymphoid cells; specifically helper T-cells at the lower dosages (15 mg/kg and 22.5 mg/kg) and cytotoxic T-cells at the high dose (30 mg/kg), and an increase in cells of myeloid lineage, specifically the monocyte/macrophage cells. These results are consistent with our histological analysis of the spleen, exhibiting primarily red pulp dysplasia.

It has been suggested that SOS-related portal hypertension is a contributing factor in oxaliplatin-induced splenomegaly [10, 11]. A previous report administering all three components of the FOLFOX regimen (leucovorin, fluorouracil and oxaliplatin) in conjunction with a specific diet (devoid of plant based materials which may act as antioxidants) demonstrated the development of SOS in mice [38]. However, mice eating a normal chow-based diet do not develop histological signs of SOS when treated with the FOLFOX regimen [38, 39]. Therefore, the lack of SOS in our oxaliplatin model is not unexpected, and the splenomegaly we observed has an aetiology independent of SOS. In conjunction with the fact that mice appeared to develop severe signs of anaemia in the extremities, and our previous findings indicating oxaliplatin-treated mice exhibit signs of fatigue such as reduced exploratory behaviour [40], the evidence indicates that the primary pathology causing splenomegaly in this model is oxaliplatin-induced haematological toxicity. These findings warrant future clinical investigation to ascertain if the incidence of splenomegaly occurring in a substantial number of CRC patients is related to the oxaliplatin-induced haematological toxicity, as observed in the mouse model reported here.

In summary, our results suggest that treatment with a high cumulative dose of oxaliplatin induces splenomegaly, abnormal spleen histology, altered spleen immune cell and cytokine profiles, and is associated with haematological toxicity and anaemia. The oxaliplatin dosage regimen and route of administration used in this study is similar to numerous other rodent studies [28, 41, 42]. Thus, the effects of splenomegaly and abnormal haematological profiles should be thoroughly considered when designing chronic oxaliplatin protocols for preclinical investigation of its anti-tumour, metabolic or neurotoxic effects. Furthermore, these systemic changes could also have wider clinical implications relating to treatment toxicity, potentially contributing to adverse side effects such as chronic fatigue, which is associated with anaemia [43, 44] and neurotoxicity, as both anaemia [45, 46] and immune response [28, 47] play a significant role in peripheral nerve damage. Similarly, the relationship between immune profiles and chemotherapy-induced peripheral neuropathy [28, 47] and between splenomegaly and oxaliplatin neurotoxicity [23] suggests that our results may have implications for the pathophysiology of oxaliplatin-induced peripheral neuropathy.
